# The multifaceted nature of plant acid phosphatases: purification, biochemical features, and applications

**DOI:** 10.1080/14756366.2023.2282379

**Published:** 2023-11-20

**Authors:** Lokesh Sharma, Amol Kahandal, Anant Kanagare, Atul Kulkarni, Chandrakant K. Tagad

**Affiliations:** a School of Bioengineering Sciences & Research, MIT Art, Design and Technology University, Pune, India; b Department of Chemistry, Deogiri College, Aurangabad, India; c Symbiosis Centre for Nanoscience and Nanotechnology, Symbiosis International (Deemed University), Lavale, India; d Department of Biochemistry, S.B.E.S. College of Science, Chhatrapati Sambhajinagar, India

**Keywords:** Plant acid phosphatases, purification, biochemical properties, applications

## Abstract

Acid phosphatases (EC 3.1.3.2) are the enzymes that catalyse transphosphorylation reactions and promotes the hydrolysis of numerous orthophosphate esters in acidic media, as a crucial element for the metabolism of phosphate in tissues. Inorganic phosphate (Pi) utilisation and scavenging, as well as the turnover of Pi-rich sources found in plant vacuoles, are major processes in which intracellular and secretory acid phosphatases function. Therefore, a thorough understanding of these enzymes’ structural characteristics, specificity, and physiochemical properties is required to comprehend the function of acid phosphatases in plant energy metabolism. Furthermore, acid phosphatases are gaining increasing importance in industrial biotechnology due to their involvement in transphosphorylation processes and their ability to reduce phosphate levels in food products. Hence, this review aims to provide a comprehensive overview of the purification methods employed for isolating acid phosphatases from diverse plant sources, as well as their structural and functional properties. Additionally, the review explores the potential applications of these enzymes in various fields.

## Introduction

Enzymes are one of the most intriguing macromolecules produced by living systems that affect biological processes. Being produced in living organisms, these proteins functioning as enzymes under physiological circumstances have a defined catalytic activity that are well known for playing a crucial role in various biological processes. Enzymes have tremendous catalytic potential for use in industry as they allow for the accelerated manufacturing of products at temperatures and pressures that are comparably lower while employing less expensive raw materials. Their remarkable potential have been employed in plentiful industrial application since over two decades. Currently, industrial biotechnology research is focused on the exploitation of novel raw materials to produce goods that are more affordable, of higher quality, include fewer toxins, and are less hazardous to the environment.

Enzymes are highly fascinating macromolecules produced by living organisms that play a crucial role in biological processes. As catalysts, enzymes exhibit well-defined catalytic activities under physiological conditions. They possess remarkable potential for application in industrial processes. Using enzymes can accelerate the formation of various products at lower temperatures and pressures while using less costly raw materials. The primary objective of industrial biotechnology is to exploit raw materials to manufacture more affordable, higher-quality products that have reduced toxicity and environmental hazards.

Industrial enzymes offer several advantages, including enhanced process efficiency, reduced energy requirements, milder reaction conditions, and improved sustainability. Their utilisation allows for the development of more environmentally friendly manufacturing processes that minimise waste generation and reduce reliance on harmful chemical agents. Additionally, the ability to utilise enzymes derived from novel sources and genetically engineered variants provides opportunities for tailored enzymatic activities, further expanding their potential applications in industry. Therefore, enzymes represent a significant asset in industrial biotechnology, and their market value continues to grow as industries recognise their immense potential for improving production processes and generating sustainable and eco-friendly products. Proteases, glycosidases, lipases, pectinases, phosphatases, and other enzyme families are particularly significant in terms of their commercial importance^1^. The valuation of the global enzymes market is projected to reach $13.2 billion by 2027 at a compound annual growth rate (CAGR) of 8% throughout the course of the period being forecast^2^. The vast need for enzymes in end-use sectors, including food and beverage, home cleaning, animal feed, and biofuel is directly proportional to the growth of the enzyme industry, which in turn is directly proportional to the growth of the enzyme market. In addition, the rising level of health consciousness among consumers is anticipated to be a primary driver of growth in the worldwide enzyme market. This, in turn, has led to an increase in the demand for functional food products^3^.

## Acid and alkaline phosphatase

Davies was the first to suggest the acid and alkaline phosphatases to identify phosphatases with significantly differing pH maxima^4^. The enzymes that catalyse the hydrolysis of phosphoric acid esters are known as phosphatases. The substrate selectivity of the two groups of enzymes also reveals differences between them. In contrast to acid phosphatase, alkaline phosphatases hydrolyse phosphorothioate acid monoesters with an S substitution but not those with an O substitution (R-SPO_3_H_2_)^5^^,^^6^.

## Acid phosphatase

Acid phosphatases, also known as orthophosphoric monoester phosphohydrolase (EC 3.1.3.2), are a class of enzymes that catalyse transphosphorylation events as well as the hydrolysis of certain orthophosphate esters in acidic media. These enzymes are known as acid phosphatases, phosphoprotein phosphatases, or ATPases because they hydrolyse a large number of phosphomonoesters with a wide range of structural variations and have a broad, overlapping substrate specificity. Acid phosphatase catalyses an apparent transition state displacement and the P-O cleavage.

They are one of the most pervasive enzymes and are abundantly present in nature. They are present in a variety of animal organs, including the kidney, liver, spleen, erythrocytes, blood plasma, seeds of higher plants, fungus, yeasts, fungi, fruits, and erythrocytes^7–13^. This widespread distribution in both lower and higher creatures raises the possibility that they play a role in fundamental biological reactions.

A particular type of phosphatases known as purple acid phosphatases (PAPs) are metalloenzymes catalysing the hydrolysis of various phosphate esters and anhydrides. Others are adept at hydrolysing phytate or 2-phosphoenolpyruvate (PEP), whereas some plant PAPs prefer ATP as the substrate^14^. Metallohydrolases constitute a substantial group of predominantly bimetallic enzymes that employ metal ions to facilitate the activation of both the nucleophile and scissile link of diverse phosphate compounds^15^.

### Acid phosphatases in plants

Acid phosphatase serves as a phosphate (Pi) scavenger in plants. When under stress, such as during a drought or a Pi shortage, they mobilise Pi to support growth^16^^,^^17^. The enzyme dephosphorylates phosphorylated protein kinases and other phosphoproteins that contain phosphorylated serine. The structure, function, catalytic mechanism, and enzyme kinetics of acid phosphatase from various plant sources are currently the subject of intensive research. These studies might be important for considering every angle in the agricultural and industrial fields^17–19^.

### Localisation of acid phosphatases in plants

The physiological function of acid phosphatases in plant metabolism and their localisation are tightly related. To further understand acid phosphatase’s precise function in plant physiology, researchers have studied its intra- and subcellular distribution^20^. For their controlled physiological effects, phosphatases are localised in certain plant cell compartments. Using electron microscopic cytochemical techniques, the intracellular localizations of plant acid phosphatases have been investigated^21^. In specific cell compartments, the phosphatases are located. In a suspension culture of black mustard cells, Stephen M. G. Duff et al. revealed that acid phosphatase was located in the cell wall^22^.

Similarly, Rumi Kaida et al. has demonstrated the location of acid phosphatase in the cell wall of tobacco cells, where the enzyme may play a role in making phosphate from the phosphoprotein casein available for cell development^23^. In Arabidopsis suspension cells and seedlings that are deficient in phosphate, acid phosphatase has been found to be localised in the vacuole^24^. Rumi Kaida et al. has demonstrated the presence of PAP in the Golgi apparatus of tobacco cells using a double-immunofluorescence labelling technique^23^.

### Functions of acid phosphatases in plants

The abundant occurrence of acid phosphatase in both lower and higher creatures raises the possibility that they are involved in the basic biological processes of organisms^13^^,^^25^. One of the most essential macro elements for the growth and development of higher plants is phosphorus, which is well known. Furthermore, it performs a crucial function as a component of macromolecules like phospholipids, proteins, and nucleic acids. It is well known that phosphatases are essential for phosphate cycling in plants. Phosphate esters’ hydrolysis is a crucial step in the metabolism of energy, the control of metabolism, and numerous cellular signalling pathways. Numerous proteins, including histones, permeases, regulatory proteins, and various enzymes, depend on the actions of protein kinases and phosphatases to function^7^. When phosphate levels are restricted, plant growth and development are significantly harmed. The majority of plant acid phosphatases lack significant substrate selectivity and have an optimum pH below 6.0. Plant acid phosphatases are typically multiform and exhibit many biochemical traits^10^.

While some acid phosphatases work to recycle Pi in the vacuoles of Pi-starved plants, others scavenge Pi from extracellular Pi-esters^26^. In order to release the free soluble form of phosphate and make it available for the range of reactions in the growing seedlings, phosphate in seeds must first be hydrolysed. The modulation of cytosolic Pi levels may involve an intracellular version of the enzyme^26^. The breakdown of organic phosphate into soluble phosphate and the efficient uptake of this phosphate by plants are both facilitated by root acid phosphatases.

Evidence shows that some plants secrete more acid phosphatases when the phosphate level is low^27^^,^^28^. It is hypothesised that intracellular and secreted acid phosphatases play a significant role in the phosphate scavenging, utilisation, and turnover of phosphate-rich sources^29^. There is no question that intracellular acid phosphatases play a role in the regular use of phosphate reserves or other compounds containing phosphate^22^. Plants have created a variety of adaptive mechanisms to improve the availability and boost the uptake of Pi in response to a persistent Pi deficit. Production and secretion of phosphatases to liberate Pi from organic forms is one example of an adaptive process. In tomatoes and other plants, higher levels of acid phosphatases have been seen at times of stress and nutrient deficiency, essentially of Pi^30^^,^^31^. According to Goldstein et al., early orthophosphate (Pi) deprivation increases acid phosphatase activity in tomatoes in whole plants and suspension-cultured cells. As a result, higher plants have a Pi starvation rescue system^30^. According to reports, salt stress raises the degree of acid phosphatase activity in sorghum and pearl millet seeds^32^^,^^33^. According to Nasri et al., during the germination stage, acid phosphatase levels in salt-tolerant varieties of lettuce rose due to the salinity of the water, whereas they fell in salt-sensitive plants. Increased phosphatase activity under salt stress conditions in resistant cultivars suggests that they directly contribute to the cells’ increased energy needs to combat the adverse effects of salinity^34^^,^^35^.

Banana (*Musa paradisiacal* L. cv. false horn) fruit was studied during ripening to determine the activity of non-specific acid phosphatases. All stages of banana ripening were found to boost acid phosphatase activity considerably. These phosphohydrolytic enzymes assist in supplying the inorganic phosphate necessary for the upkeep of numerous cellular metabolisms, such as the conversion of starch to sugar and the resulting increase in respiration that take place during fruit ripening^36^.

Dodd et al. measured the levels of acid phosphatases in the roots and rhizosphere of rape, wheat, and onion When infected with vesicular-arbuscular mycorrhizal fungi^37^. When compared to uninfected plants, they discovered higher amounts in the roots and rhizosphere of infected plants. Phytases, phosphoglycolate phosphatases, phosphotyrosyl protein phosphatases, ATPases, and other intracellular acid phosphatases belong to a distinct class that exhibits substrate selectivity^22^. These acid phosphatases perform several metabolic tasks. The PAPs in red kidney beans prefer ATP over other substrates, indicating that they may function as an ATPase during seed germination^38^. This enzyme’s activity increased during senescence at the start of wilting^39^.

Along with the acid phosphatases from poppy seeds, wheat, maize seedlings, and other sources, potatoes exhibit significant activity and specificity for phosphorylated tyrosine (P-Tyr)^29^. As a result, it is possible that the enzyme could act in vivo as a P-Tyr protein phosphatase. In non-plant species, phosphotyrosyl protein phosphatases (EC 3.1.3.48) have been linked to cell cycle regulation and cellular differentiation. However, relatively little is known regarding the functions or mechanisms of Tyr-phosphorylation in plants.

### Assay methods

**Figure 1.**
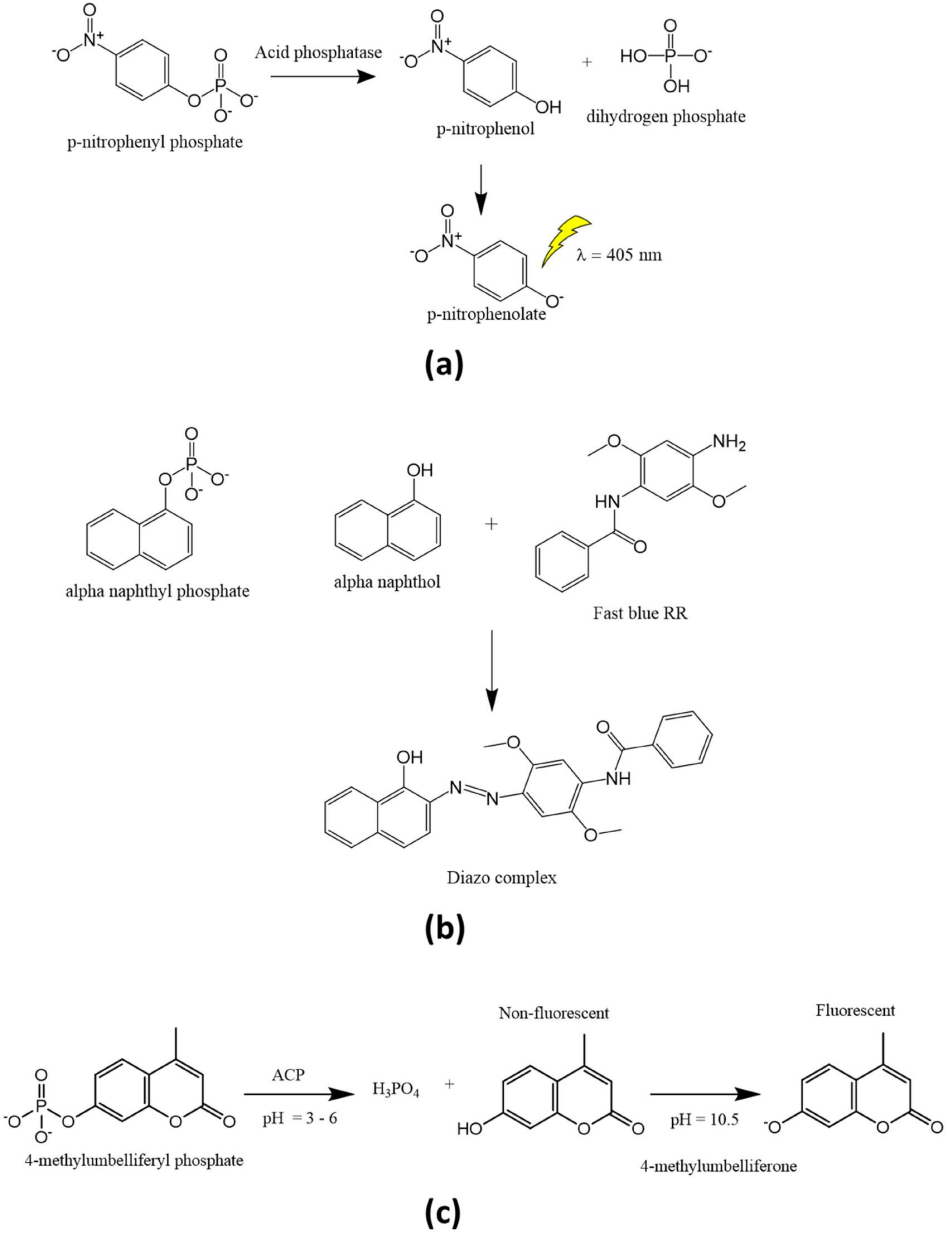
Determination of acid phosphatase activity using (a) p-nitrophenyl phosphate, (b) alpha-naphthyl phosphate, and (c) 4-mrthyllumbelliferyl phosphate substrates.

Acid phosphatase activity can be estimated by using a variety of substrates. The enzymatic reactions using the p-nitrophenyl phosphate, α-naphthyl phosphate and 4-methylumbelliferyl phosphate are depicted in Figure 1. P-nitrophenyl phosphate is the substrate used most frequently by researchers to measure the activity of acid phosphatase. Hydrolysis of p-nitrophenyl phosphate by acid phosphatase results in the formation of p-nitrophenol and, subsequently, p-nitrophenolate upon the addition of NaOH (0.5–1 N) to the reaction mixture (Figure 1(a)). By using spectrophotometry at 405 nm, it is possible to measure the rate of p-nitrophenol release^11^^,^^40^^,^^41^.

**Figure 2.**
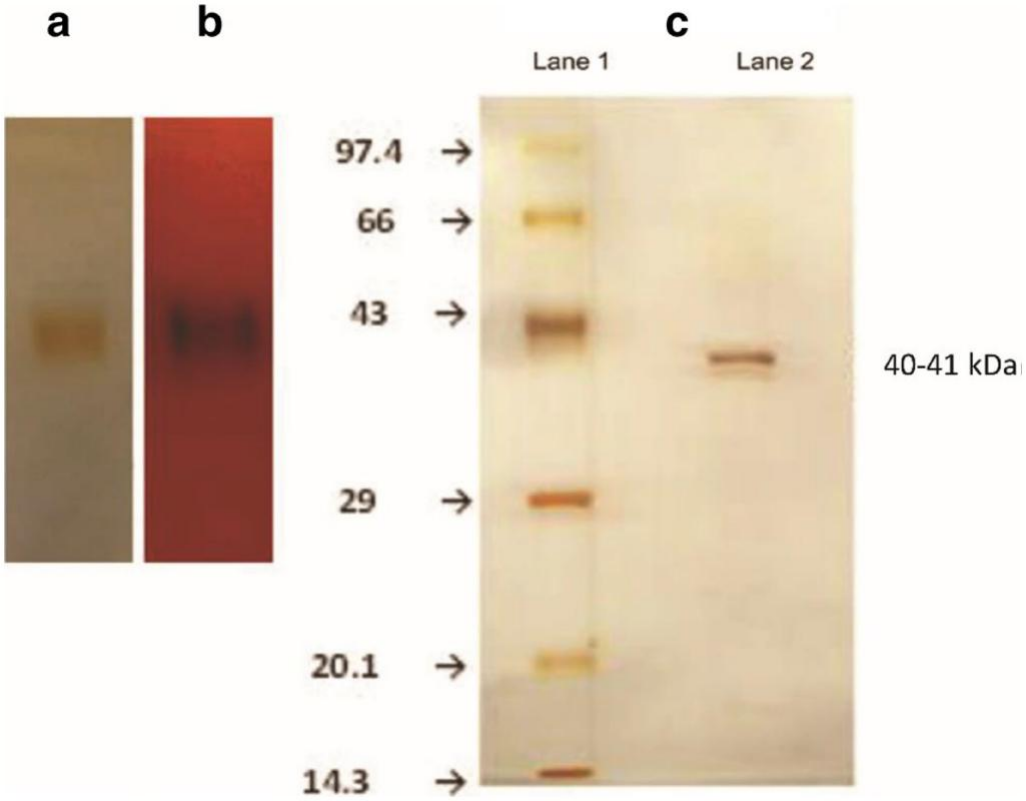
(a) Native PAGE of rice bean acid phosphatase stained with silver staining showing a single band. (b) Activity-stained gel using Fast Garnet GBC salt and α-naphthyl phosphate. (c) Lane 1 Mobility of silver stained standard molecular mass marker proteins (14.3–97.7 kDa). Lane 2 Denaturing SDS-PAGE of silver stained rice bean acid phosphatase^55^.

Acid phosphatase is prone to surface inactivation, which is one of the main issues with any assay for the enzyme. If there is significant phosphotransferase activity, there are differences in the amounts of phenol freed from phenolic phosphates and inorganic phosphate. The use of 4-methylumbelliferyl phosphate (Figure 2(c)) and α-naphthyl phosphate as substrates have allowed for the development of fluorogenic tests with extremely high sensitivity^6^^,^^42^. The product of enzymatic hydrolysis of α-naphthyl phosphate, i.e. α-naphthol forms diazo complex with fast blue RR indicator to give reddish coloured complex, indicating the activity of the phosphatase^43^^,^^44^ (Figure 1(b)). By measuring the rate of orthophosphate liberation, the enzyme activity for several additional phosphorylated substances has been identified (Chan et al., Ames et al., Lowry and Lopez et al., ChandraRajan and Klein et al., etc.)^45–47^.

Due to the non-specific nature of acid phosphatase, Sadia Nadir et al. used a variety of substrates while working with acid phosphatase from germinating *Vigna radiata*, including -glycerophosphate, ATP, ADP, AMP, glucose-1-phosphate, glucose-6-phosphate, fructose-6-phosphate, phenyl phosphate, and ribulose-5-phosphate^12^. Acid phosphatase from *Macrotyloma uniflorum* seeds was tested on various substrates such as ATP, ADP, glycerophosphate, and others^43^. The amount of phosphate released was taken as a measure of enzyme activity. Thus the inorganic phosphate released was measured by the method developed by Lowry and Lopez 1946^47^. Briefly, the enzymatic reaction was stopped by adding 1 ml of 3% ammonium molybdate (in 200 mM of acetate buffer, pH 4.0), followed by the addition of 0.1 ml of 1% ascorbic acid (in 200 mM of acetate buffer, pH 4.0). After 30 min of colour development, the absorbance at 700 nm was measured. A molar extinction coefficient of 4000 M^−1 ^cm^−1^ was used to quantify the amount of inorganic phosphate that was generated^43^.

### Acid phosphatase isolation and purification

Acid phosphatase has been purified to homogeneity from diverse sources, including microorganisms, plants, and animals^48–50^. Affinity chromatography using a Con-A Sepharose affinity matrix has proven effective for acid phosphatase purification from *Vigna radiata* seeds and other glycoprotein-rich samples^12^. Acid phosphatase from germination-ready seeds of *Vigna radiata* and Cucurbitaceae was isolated using hydrophobic interaction chromatography^12^^,^^49^.

Various chromatographic techniques have been employed to separate acid phosphatases from different sources, such as dry seeds, germinating seeds, and cotyledons. Koffi D. KM purified two non-specific acid phosphatase isoforms from Cucurbitaceae seeds. The purification process involved a combination of ammonium sulphate fractionation, chromatography on Sephacryl-S 100 HR, CM-Sepharose CL-6B, and Phenyl Sepharose 6 Fast Flow columns, resulting in a purification factor of 93.14^49^. Sing and Luthra isolated acid phosphatase from *Psoralea corylifolia* cotyledons. The isolation technique encompassed gel filtration, ion exchange chromatography, and ammonium sulphate precipitation^48^.

Furthermore, acid phosphatase from black gram (*Vigna mungo L*.) seedling germination was purified by Asaduzzaman et al. The purification procedure involved ammonium sulphate fractionation, gel filtration chromatography on Sephadex-G-75, and ion exchange chromatography on a DEAE cellulose column, resulting in a purification factor of 43.8^50^. Heat-stable acid phosphatase from chickpeas was also purified to homogeneity using ethanol precipitation, ion exchange chromatography on DEAE cellulose, gel filtration on Sephadex G-150, and subsequent ethanol chromatography^51^. Three isoforms of acid phosphatases were obtained from five-day-old peanut seedlings using sequential chromatography on DEAE-sepharose CL-6B, CM-sepharose CL-6B, Sephacryl S-200 HR, and Phenyl sepharose HP columns, achieving apparent homogeneity. The purification of PAP from *Euphorbia characias* latex involved protein extraction in acetone, ammonium sulphate fractionation, ion exchange chromatography on DEAE cellulose, and gel filtration chromatography with Sephadex G-200^52^. Lastly, acid phosphatase from banana fruits was isolated through hydrophobic chromatography on a Butyl-Sepharose column, ion exchange chromatography on a Fractogel EMD SO3-650 (S) cation exchange resin, and gel filtration chromatography on Superdex G-200 HR 16/60^53^. The coupling of ConA to Seralose 4B helped in the separation of acid phosphatase from the cotyledon of *Erythrina indica* based on the affinity interactions^54^.

The appearance of the single band in the Polyacrylamide gel electrophoresis (PAGE) electrophoresis is taken as a measure of the purity of the enzyme. The activity staining is usually performed to confirm that the band that appeared in the PAGE is of the phosphatase using α-naphthyl phosphate as a substrate and Fast Garnet GBC salt or Fast Blue RR salt as a chromogenic indicator which forms a coloured complex with the α-naphthol, a product of the enzymatic reaction. The appearance of the coloured band indicates that the protein is acid phosphatase. The native non-denaturing PAGE of rice bean acid phosphatase showing the silver-stained gel and the activity staining using α-naphthyl phosphate as a substrate and Fast Garnet GBC salt system is shown in Figure 2. The figure also depicts the molecular weight determination of the enzyme using a standard molecular mass marker proteins ladder.

### The biochemical characteristics of acid phosphatases

The characterisation of acid phosphatases from various sources has been the subject of extensive research. Table 1 summarises some of the characteristics of plant acid phosphatases.

**Table 1.** Biochemical characteristics of acid phosphatases purified from different plant sources.

Enzyme source	Molecular weight	Temp opt	pH opt	Activator	Inhibitor	Ref.
*Vigna radiata*	Two isoforms ∼29 kDa & ∼18 kDa	50 °C (for 29 kDa isoform)	5.5	Ca, Na	Hg, Cu, Pi, vanadate, Fluoride	^12^
*thaliana* seedlings	Native 100 (Homodimer) Subunits 55 kDa	–	5.6	Mg, Co, Mn, Ba, Mg	Fe^2+^, Cu^2+^, Zn^2+^	^24^
*Solanum tuberosum* tuber	Native 100 kDa Subunits: 55 & 57 kDa	–	5.8	Mg	molybdate, vanadate, and Zn	^29^
Cotyledons of *Psoralea corylifolia L.*	Two isoforms: ∼28 kDa & ∼30 kDa	50 °C	5.5		Cu^2+^ , NaF, NaMoO_4_	^48^
Cucurbitaceae seeds	Two isoforms: ∼70 kDa ∼55 kDa	60 °C 55 °C		Mg, K, EDTA	Zn^2+^	^49^
*V. Mungo* L. seedlings	25 kDa	55 °C	5.0	K, Cu, Ba	Mg^2+^, Zn^2+^, EDTA	^50^
*Cicer arietinum*	–	50 °C	5.5	Co^2+^, Ca^2+^	Zn^2+^, Hg^2+^	^51^
*Arachis hypogaea seedlings*PI, PIIa & PIIb	PI = 25.3 (heterodimer) Subunits: 14 and 12 kDa PIIa= 22.4 & PIIb = 24 kDa	55 °C	5.0	–	–	^52^
Banana Fruit	40 kDa	–	5.8	Mg, Mn	Pi, Mo, vanadate, arsenate,Zn^2+^	^56^
*L. esculentum*	Native 142 (heterodimer) Subunits: 63 & 57 kDa	–	5.1	Mg	Zn, Cu, Fe, molybdate, vanadate, fluoride and Pi.	^57^
Garlic seedlings	58 kDa	50 °C	5.7	Na, Ca, K	Cu, Mo, Mn	^58^
Soybean seeds	51, 58, 52 & 30 kDa for AP1, AP2, AP3A, AP3B isoforms respectively	60 °C for AP1, AP2, AP3B & 50 °C AP3A.	–	–	Pi, Fluoride, Mo, vanadate, Cu, Zn	^59^
*Carthamus oxyacantha*seedlings	36 kDa	50 °C	5.0			^60^
*Vigna umbellata* Thunb.	80 kDa.	60 °C	5.5	–	–	^55^
*Erythrina indica*	89 kDa, two subunits with MW of 38 and 42 kDa	30–50 °C	5	Zn, Hg	Cu, Ca	^54^
*Opuntia megacantha Salm-Dyck*	44 kDa	60 °C	5.5	Ca^2+^	Strongly inhibited Cu^2+^ and Fe^3+^, moderately inhibited by Mg^2+^ and Zn^2+^. Br^−^, CN^−^, F^−^, I^−^ and N^3−^	^61^
*Chenopodium murale* seedlings	29 kDa	50 °C	5	–	–	^62^
*Macrotyloma uniflorum* seeds	55 kDa, heterodimer of subunits having MW 27 and 28 kDa	50 °C	5	Mg^2+^	Strongly inhibited by Hg^2+^ moderate inhibition was observed with Ag^+^	^43^

#### Molecular weight and subunit makeup of acid phosphatases

There is a large amount of variation among plant acid phosphatases, both in terms of the molecular weight and the subunit makeup. Acid phosphatases found in a wide variety of plant sources have a molecular weight that ranges between 25 and 130 kDa, whereas acid phosphatases found in animal sources have a molecular weight that ranges between 35 and 40 kDa. Some plant acid phosphatases can exist as either homodimers or heterodimers. Homodimers have two subunits with the same amount of molecular weight, whereas heterodimers have two subunits with varying amounts of molecular weight. Each subunit of the homodimer 130 kDa acid phosphatase that is derived from *Euphorbia characias* comprises one Fe (III) and one Zn (II) ion, according to research^53^. Various organisms, including *allium sepa* bulbs, red kidney beans, barley roots, and thaliana seedlings, are found to have homodimeric acid phosphatases^24^^,^^38^^,^^56^^,^^57^. It has been discovered that many different plants can produce heterodimeric acid phosphatases. One example is the acid phosphatases found in potato tubers, which comprise 57 kDa and 55 kDa subunits^29^. It was observed that the major isoform of acid phosphatase in peanut seedlings is a heterodimer consisting of 14 kDa and 12 kDa subunits of the enzyme^52^. There is evidence of the presence of the 31 kDa and 28 kDa subunits of soybean root nodule acid phosphatase^58^. The acid phosphatase produced by tomato plants that are deprived of phosphorus is a heterodimeric protein composed of two 57 and 63 kDa subunits^59^. The major isoform of acid phosphate purified from the seeds of *Macrotyloma unniflorum* seeds sourse has a molecular weight of 55 kDa with the presence of heterodimers of molecular weight of 27.093 and 28.241 kDa as identified with MALDI-TOFF analysis^43^. The extraction of acid phosphatase from *Vigna mungo* seedlings revealed the presence of a single acid phosphatase with a molecular weight of 25 kDa^49^. Additionally, it has been discovered that several acid phosphatases have various subunit compositions. Research has shown that cotton seedling acid phosphatase is a tetrameric protein with a total molecular weight of 200 kDa and comprises four subunits, each with a size of 55 kDa^60^.

#### Isoforms of acid phosphatases

It has been discovered that distinct plant tissues have multiple varieties of acid phosphatases. Various variables, including physiological and environmental ones, control the expression of these different forms of acid phosphatase^61^. Using a Sephadex G-100 column for separation, Asahi et al. and Uehara et al. found evidence of the presence of at least five unique isoforms of acid phosphatase in sweet potatoes^62^^,^^63^. This was demonstrated by the detection of two peaks during the separation process. In a similar vein, it was discovered that potatoes contain six distinct isoforms of acid phosphatase, each of which has a different amount of glycosylation^29^. It was found that the seeds of plants belonging to the Cucurbitaceae family have two distinct kinds of acid phosphatase with molecular weights of 70 and 55 kDa, respectively^49^. The cotyledons of *Psoralea corylifolia* showed evidence of the presence of five different isoforms of the acid phosphatase enzyme^49^. It was discovered that the root nodules of soybeans have three isoforms of the enzyme acid phosphatase, and it was also shown that peanut seedlings have the same number of isoforms^52^^,^^58^. In general, acid phosphatase can be found in four different isoforms. These isoforms have molecular weights ranging from 51 to 58 kDa and are designated AP1, AP2, AP3A, and AP3B, respectively^64^. Three isoforms of acid phosphatase were detected in the *Macrotyloma uniflorum* seeds^43^.

#### Metal concentration, colour, and visible absorption spectrum

The classification of acid phosphatases as metalloenzymes was uncovered through research on acid phosphatases derived from various sources. The source organism determines the particular metal ion (Mn or Fe) which is needed for the enzymatic activity of acid phosphatases. Plant PAPs almost always have a Fe (III)-X(II) active site, where X can stand for either Zn^2+^ or Mn^2+^. These enzymes exhibit a discernible purple colour as a result of a charge transfer transition occurring between a tyrosine ligand located at the active site and a Fe(III) ion^15^^,^^28^^,^^65–67^. Analysis of the red kidney bean acid phosphatase using atomic absorption spectra revealed the presence of iron and zinc elements but not manganese. Additional research using X-ray absorption techniques verified the presence of a binuclear Fe(III)-Zn(II) core in red kidney bean acid phosphatase^38^^,^^68^. It has been hypothesised that rice, soybeans, spinach, and the tubers of sweet potatoes all have Mn^2+^-containing PAP in their cell walls^6^^,^^69–71^. However, studies carried out by Hefler and colleagues found that sweet potato acid phosphatase primarily includes Fe atoms and very insignificant levels of Mn. This indicates that Fe, rather than Mn, is likely involved in catalysis and the visible chromophore of the enzyme^70^. Compared to copper and zinc, the concentration of manganese in soybeans was significantly greater. This finding suggests that manganese and/or iron play a substantial role in the effective binding of phosphate and the acceleration of phosphomonoester hydrolysis^71^.

In addition, PAPs have conserved structures in both their catalytic sites and domains^65^^,^^67^. This is confirmed by the highly conserved motifs located inside the catalytic domains and the alignment of metal ligating residues^65^^,^^72^. There are variances in the oligomeric architectures of PAPs found in bacteria, mammalian organisms, and plant life, despite the fact that conservation of their catalytic domain^28^^,^^65^^,^^67^. Charge transfer changes from metal-coordinating Tyr to the metal-ligand Fe (III) at around 560 nm contribute to the appearance of PAPs in the solution, giving these enzymes their distinctive colours, which can range from pink to purple^28^^,^^67^. This phenomenon is responsible for their visual appearance. According to the findings of several different investigations, the natural colouring of these enzymes is thought to be caused by the presence of metal ions. For example, the colour of sweet potatoes is assigned to Mn(III)-phenolate bonds, but the colour of red kidney beans is related to charge transfer transitions between Fe(III)-phenolate and phenolate^73^. Similarly, the colour of purple sweet potatoes is attributed to Mn(III)-phenolate bonds^38^.

**Figure 3.**
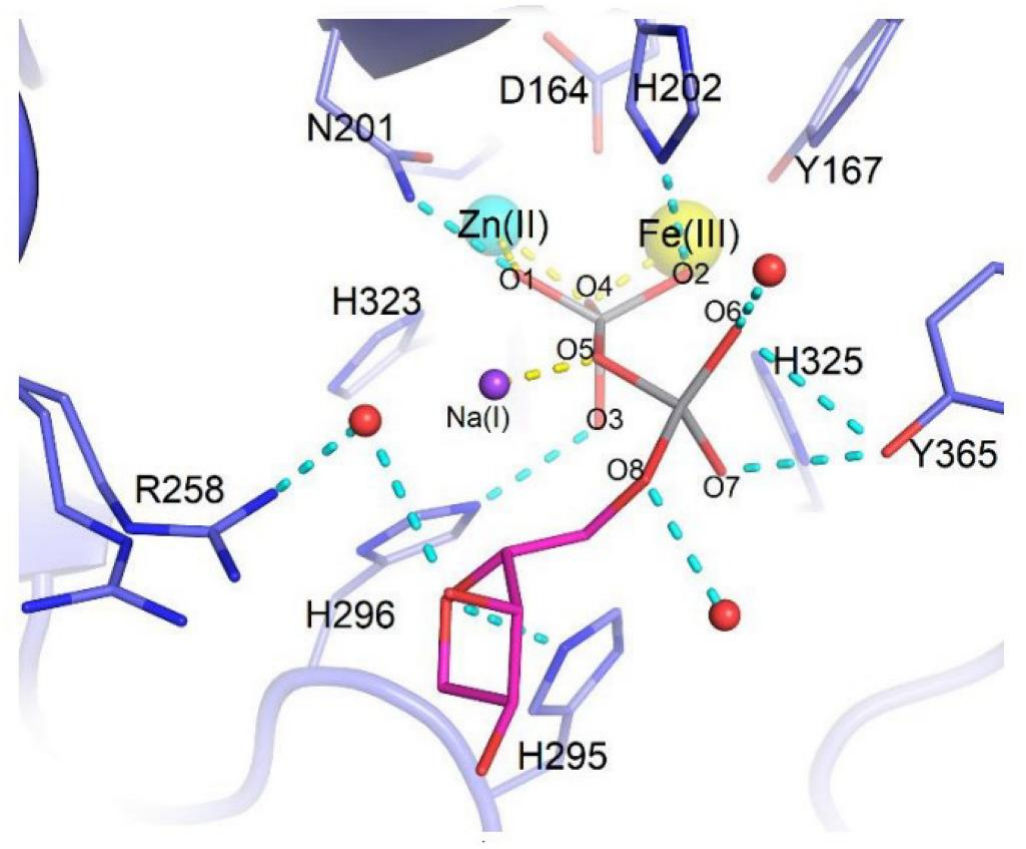
Stick representation of ADV (magenta carbons) bound to the active site of rkbPAP (light blue carbons). The metal ions are shown as spheres with Fe(III) in yellow, Zn(II) in cyan and Na(I) in purple. Bonds to metal ions are in yellow and hydrogen bonds are in light teal. Water molecules are shown as red spheres. Copyright @ Wiley, Feder et al.^15^.

The catalytic mechanism of these enzymes involve five-coordinate oxyphosphorane species in the transition state^74^. Feder et al. crystallised PAP from red kidney beans (rkbPAP), wherein both adenosine and vanadate were present. The vanadate analogue of ADP, which was synthesised *in crystallo*, offers a comprehensive understanding of the binding mechanism of a phosphatase substrate^15^. This structure accurately represents the hypothetical five-coordinate transition state. The present study holds considerable importance as it offers a credible representation of an enzyme substrate complex during its associative transition stage. The structure described in this study supports the probable reaction mechanism utilised by PAPs and similar metallohydrolases. The angle of 168.19° observed in the O4-VO5 axis (Figure 3) provided evidence for a model proposed by Feder et al. in which the nucleophilic O4 atom, situated in a bridging position between the two metal ions, forms a bond with the phosphorus atom of the substrate molecule. This interaction results in the cleavage of the opposing bond (represented by V-O5) and the subsequent liberation of the leaving group^15^.

There have been many reports of broad absorption ranges in the 510–560 nm region in relation to the visible absorption spectra of acid phosphatases. The spectrum of red kidney bean phosphatase peaks at 540 nm^69^, whereas the spectrum of soybean phosphatase reaches 560 nm^38^^,^^68^. Sugiura et al. show that sweet potatoes have a significant absorption band of about 515 nm^22^. On the other hand, acid phosphatase from the same source exhibits a broad peak at roughly 555 nm, as stated by Uehara et al.^37^ Acid phosphatase has a maximal absorption at 510 nm in its catalytically active reduced form, which is pink in colour. However, acid phosphatase exhibits absorption maxima at 550 nm in its oxidised state, which is catalytically inactive and purple. Acid phosphatase has absorption maxima at 510 nm in its reduced form.

#### Optimum temperature

A certain temperature is required for enzymes to function at their highest level of catalytic activity. At the optimal temperature, the structure of the active site of enzymes remains more stable, which enables a more effective conversion of substrate to product. As seen in Table 1, acid phosphatases obtained from various sources have a wide range of optimal temperatures and varying degrees of stability when exposed to heat. A sizeable portion of the acid phosphatases that have been characterised come from various sources and exhibit remarkable thermal stability. The enzymatic activity of acid phosphatase extracted from *Psoralea corylifolia L*. cotyledons and garlic seedlings was shown to be most effective at approximately 50 °C^48^^,^^75^.

Similarly, the ideal temperatures for the acid phosphatase isoforms extracted from the Cucurbitaceae family seeds were 60 °C and 55 °C, respectively^49^. The optimum temperature for the activity of chickpea acid phosphatase and the primary isoform of acid phosphatase from *Vigna radiata* was found to be 50 °C^12^^,^^51^. At a temperature of 55 °C, acid phosphatase from *Vigna mungo L*. and all three acid phosphatase isoforms derived from peanut seedlings showed their highest activity levels^50^^,^^52^. In the instance of *Pinctata Fucata*, the isoforms of acid phosphatase I and acid phosphatase II showed the highest levels of enzyme activity at temperatures of 47 °C and 57 °C, respectively^76^.

#### Optimum pH

In order to catalyse their respective functions, the vast majority of acid phosphatases require an acidic environment. However, the pH that acid phosphatase from different sources ideally should be at in an acidic medium is presented in Table 1 below. Acid phosphatase from cotyledons of *Psoralea corylifolia L.* has the highest levels of enzyme activity at pH 5.5 and pH 5.7, respectively^48^^,^^75^. It was discovered that the two acid phosphatase isoforms from Cucurbitaceae seeds have a pH optimal of 5.6^49^. At a pH of 5.5, it was found that chickpea acid phosphatase and high molecular weight isoforms of *Vigna radiata* had the highest levels of activity^12^^,^^51^. Optimum pH of acid phosphatase from *Carthamus oxyacantha* seedlings, *Opuntia megacantha Salm-Dyck*, *Chenopodium murale* seedlings and *Macrotyloma uniflorum* seeds was found to be 5^43^^,^^77–79^.

On the other hand, acid phosphatase from *Vigna mungo* L. had the lowest levels of activity at this pH. At a pH of 5.0, the seedling enzyme activity was at its highest^50^. All three isoforms of the acid phosphatase found in peanut seedlings reached their maximum activity level when the pH was adjusted to 5.0^52^. Both acid phosphatase I and II, isoforms from the *Pinctata Fucata*, had the maximum levels of enzyme activity at a pH of 4.6 and 3.2, respectively^76^.

#### Substrate specificity

Acid phosphatases are enzymes that may hydrolyse a large variety of phosphomonoesters with various structural variants. They are also referred to as phosphoprotein phosphatases or ATPases. They have overlapping and extensive substrate specificity. Based on their preferred substrates, plant acid phosphatases can be divided into specialised and non-specialised acid phosphatases^22^. Non-specialised acid phosphatases lack unique preferences for any one substrate, whereas specialised acid phosphatases show distinct but not rigid substrate preferences. Numerous naturally occurring and synthesised phosphorylated substances are hydrolysed by acid phosphatases found in various sources. P-nitrophenyl phosphate is a typical synthetic substrate for acid phosphatases. Additionally, 4-methylumbelliferyl phosphate and α-naphthyl phosphate, which are fluorescent substrates, are hydrolysed by acid phosphatases^6^^,^^42^.

In many biological systems, phosphorylated compounds serve important roles. Phosphorylated sugar molecules, nucleotides, and phosphorylated amino acids are just a few of the things that acid phosphatases break down. According to reports, when Glycine max seeds germinate, acid phosphatase activity is seen towards a variety of substrates^11^. Among these substrates, pyrophosphate (PPi) was the one where the enzyme was most active. It has also shown activity towards phosphoenol pyruvate (PEP), glucose-6-phosphate, and tyrosine phosphate (TyrP), albeit to a lesser amount^11^. Studies on the acid phosphatase in banana fruit have shown that it prefers phosphoenol pyruvate, O-phospho-L-tyrosine, PPi, as well as other tri-, di-, and phenyl phosphates as substrates^80^. High specificity was demonstrated by recombinant kidney bean acid phosphatase when hydrolysing PPi, phosphoenol pyruvate, and phosphorylated-Ser, -Thr, and -Tyr substrates^81^.

## Applications of acid phosphatases

**Figure 4.**
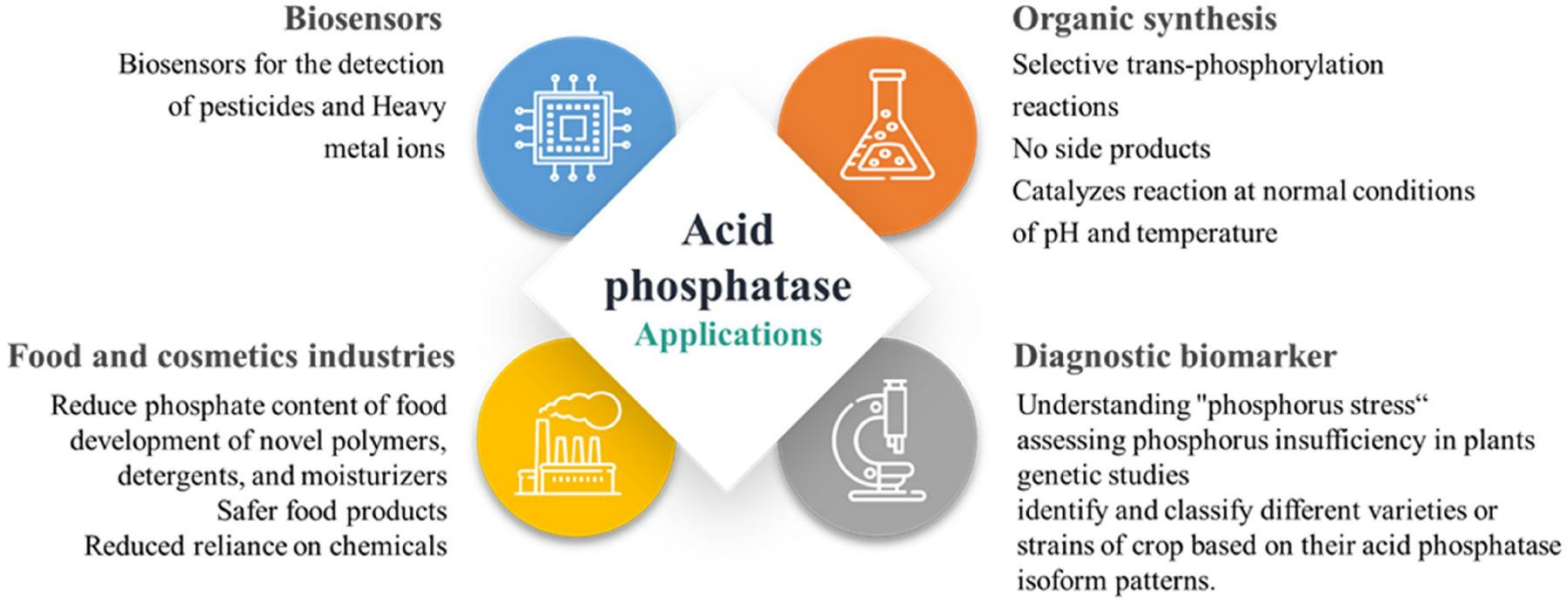
Applications of acid phosphatases.

Enzymes are widely utilised in various industrial applications due to their effectiveness, selectivity, and ability to accelerate reactions. The advancements in enzyme biotechnology have ushered in a new era of enzyme applications in industrial processes. Proteases, glycosidases, lipases, phosphatases, and other enzymes belong to the primary category of enzymes that hold great significance for industrial purposes. The applications of acid phosphatases in various fields are summarised in Figure 4.

### Applications in industry

Enzyme-based phosphorylation technology is increasingly being applied across various sectors^19^^,^^82^. Acid phosphatase, for example, is utilised to decrease the phosphate content in caseins, which predominantly consist of phosphoserine residues, thereby reducing phosphate toxicity. In the food industry, acid phosphatases are employed to produce phosphorylated substances that serve as flavour enhancers or nutritional supplements^19^.

Furthermore, acid phosphatases find applications in the development of novel polymers, detergents, and moisturisers that incorporate cosmetic ingredients^19^.

### Acid phosphatase as a diagnostic tool

Acid phosphatases in plant tissues can provide valuable insights into understanding "phosphorus stress" and assessing phosphorus insufficiency in plants. Phosphorus is an essential nutrient for plant growth and development, and its availability can significantly impact plant health and productivity. By examining the levels of acid phosphatases in plant tissues, researchers can gain insights into the plant’s response to phosphorus stress. Phosphorus stress refers to the inadequate availability or inefficient uptake of phosphorus by plants, which can limit their growth and development. Acid phosphatases play a crucial role in phosphorus acquisition and metabolism in plants.

In genetic studies on rice germplasm, acid phosphatases in the leaf tissue have been used as biochemical markers. These markers help researchers identify and classify different varieties or strains of rice based on their acid phosphatase isoform patterns. By studying the variations in acid phosphatase isoforms, researchers can better understand the genetic diversity and characteristics of different rice germplasm lines. This information is valuable for plant breeders and geneticists developing improved rice varieties with enhanced phosphorus utilisation efficiency or tolerance to phosphorus deficiency. By utilising acid phosphatases as biochemical markers, researchers can make informed decisions regarding the selection and breeding of rice plants with desirable traits related to phosphorus uptake and utilisation^27^.

### Acid phosphatases in the synthesis of organic compounds

Enzymatic phosphorylation plays a critical role in natural systems, involving the addition of a phosphate group to a molecule. Acid phosphatases are utilised to facilitate phosphorylation reactions through enzymatic pathways^82^^,^^83^. Phosphate esters hold significant biological and industrial importance, leading to the development of various chemical and biochemical phosphorylation methods^82^. Traditional chemical procedures often involve the use of strong chemicals that can modify additional functional groups on molecules, necessitating the use of protective group chemistry^82^^,^^83^. However, the application of phosphatases can overcome these limitations due to their stereo and regio-selective nature. Employing phosphatases to phosphorylate polyhydroxy compounds simplifies the process, enhances practicality, and reduces complexity. Furthermore, enzymes operate under mild conditions and typically generate less waste^82^. The acid phosphatase derived from *Shigella flexneri* can catalyse transphosphorylation, involving the transfer of phosphate groups from PPi to alcohol groups^84^. *Shigella flexneri* acid phosphatase exhibits regiospecificity (primarily phosphorylates primary alcohols), exceptional stability, and a broad optimal pH range, enabling its utilisation at higher alkaline pH values^82^. The utilisation of phosphorylated intermediates is crucial in multi-enzyme cascade reactions^82^.

Researchers have developed a continuous-flow reactor for the enzymatic production of phosphorylated compounds^82^. Immobilising acid phosphatase on solid support enables cost savings, increased enzyme stability, and catalyst recycling. Using the immobilised acid phosphatase in either a fed-batch reactor or a packed-bed continuous reactor, several primary alcohols were successfully phosphorylated in the presence of PPi. Covalent attachment of the enzyme to the beads allows for the physical separation of phosphorylated products from the enzyme, preventing hydrolysis. Acid phosphatases have been employed to convert various compounds, including D-glucose, N-acetyl-D-glucosamine, dihydroxyacetone, glycerol, rac-glycerol-1-phosphate, allyl alcohol, allyl phosphate, inosine, and inosine-5′-monophosphate^82^. In the synthesis of compounds of biological interest, acid phosphatases are frequently utilised for selective dephosphorylation. For instance, acid phosphatase was used for the selective dephosphorylation of 2-carboxy-D-arabinitol 1,5-bisphosphate to produce 2-carboxy-D-arabinitol-1-phosphate, a naturally occurring inhibitor of ribulose 1,5-bisphosphate carboxylase^6^.

### Acid phosphatase as a biosensor: applications

An amperometric biosensor utilising acid phosphatase inhibition has been reported to detect arsenic (V)^85^. The enzyme was cross-linked and immobilised on screen-printed carbon electrodes (SPCEs). The presence of arsenic (V) ions decreased the amperometric response to the substrate 2-phospho-l-ascorbic acid. The sensor exhibited a detection limit of 0.11 M and successfully determined the arsenic (V) amount in groundwater samples^85^. The immobilisation of acid and alkaline phosphatases in electrodes enabled the potentiometric detection of pesticides^86^. The system consisted of three pH electrodes with ion-sensitive regions connected to a membrane containing immobilised enzyme. Changes in pH were induced by the reaction of the immobilised enzyme with the corresponding substrate before and after incubation. The ratio of potential changes in the presence and absence of pesticides was used to calculate pesticide inhibition^86^.

A biosensor was developed using acid phosphatase and glucose oxidase to detect organophosphate pesticides^87^. Both enzymes were immobilised on different dialytic membranes using a poly azetidine prepolymer as the immobilising agent. The dual enzyme electrode system involved the continuous oxidation of glucose 6-phosphate by glucose oxidase, which, in the presence of acid phosphatase, converted to glucose and produced gluconic acid. The activity of acid phosphatase was suppressed by organophosphate pesticides, resulting in a decrease in the amperometrically measured quantity of H_2_O_2_ using the dual enzyme electrode^87^. Furthermore, a rapid and easy-to-use biosensor for detecting Hg^2+^ was developed based on inhibiting enzyme activity using acid phosphatase from *Macrotyloma uniflorum* seeds^88^.

### Inhibitor development

A growing number of members of the large family of binuclear metallohydrolases have also emerged as targets for chemotherapeutics to treat a diverse range of human disorders^89^. Specifically, elevated levels of PAP activity in osteoclasts are indicative of an osteoporotic phenotype and consequently human PAP is a current target for the development of novel drugs to treat osteoporosis^89^^,^^90^. Using a combination of fragment-based screening and rational design inhibitors for PAP have been developed with inhibition constants in the nM range^89^^,^^90^. These inhibitors are selective for the active site of PAPs but are not yet soluble enough to be considered as suitable drug leads.

## Conclusion

In conclusion, acid phosphatases (EC 3.1.3.2) perform crucial roles in both the phosphate metabolism in tissues and the turnover of Pi-rich sources in plant vacuoles. Their capacity to catalyse transphosphorylation reactions and hydrolysis of orthophosphate esters in acidic environments renders them indispensable enzymes in various biological processes. This article has provided a comprehensive overview of the purification techniques used to isolate acid phosphatases from multiple plant sources, casting light on their structural and functional properties. Knowing their specificity and physiochemical characteristics may help understand their function in plant energy metabolism. In addition, the role of acid phosphatases in minimising phosphate levels in food products increases their importance in industrial biotechnology. Due to the ever-increasing interest in sustainable practices and resource management, acid phosphatases have considerable potential in numerous sectors, including agriculture, biotechnology, and food processing. Utilising the unique properties of these enzymes could result in the development of novel applications, such as enhancing Pi utilisation and scavenging in plants and creating novel techniques for reducing phosphate levels in dietary products. The additional research and investigation of their structural and functional properties will advance our understanding of plant energy metabolism and open up new avenues for their industrial application.
